# New Flexible Flame Retardant Coatings Based on Siloxane Resin and Ethylene-Vinyl Chloride Copolymer

**DOI:** 10.3390/polym8120419

**Published:** 2016-12-02

**Authors:** Dorota Wesolek, Ryszard Gasiorowski, Szymon Rojewski, Judyta Walentowska, Rafał Wojcik

**Affiliations:** Department of Innovative Biomaterials and Nanotechnologies, Institute of Natural Fibres & Medicinal Plants, Wojska Polskiego 71b, 60-630 Poznan, Poland; ryszard.gasiorowski@iwnirz.pl (R.G.); szymon.rojewski@gmail.com (S.R.); judyta.walentowska@iwnirz.pl (J.W.); rafal.wojcik@iwnirz.pl (R.W.)

**Keywords:** flame retardant, back-coating, flammability, upholstery fabrics

## Abstract

This work presents the effectiveness of a phosphorus-containing flame retardant based on siloxane resin and ethylene-vinyl chloride copolymer as a back-coating of fabrics. The possibility of improving flame retardant efficiency of this composition by introducing fumed silica, montmorillonite, carbon nanotubes, and graphite was evaluated. The effect of each additive on the efficiency of the composition was examined separately. Flammability tests of flame retardant-coated fabrics (natural and synthetic) were carried out using pyrolysis combustion flow calorimetry (PCFC), cone calorimetry, and limiting oxygen index determination. An assessment of the ignitability of upholstered furniture containing flame retardant fabric, resistance to washing, antifungal activity, and some of the utility properties of the final newly-developed flame-retardant coating was conducted.

## 1. Introduction

Textiles, thanks to their usability, decorative qualities, and the diversity of design, are widely used in residential interiors. Apart from the their undoubted advantages the main disadvantage is their susceptibility to flame and rapid spread of flames, which is a cause of many fires leading to considerable loss of life and destruction of buildings. A major threat is upholstered furniture, where the main sources of ignition are cigarettes and matches. A typical furniture fire, caused by a cigarette at the beginning, is not a rapid process. Usually, this type of fire at the smoldering phase runs for a long period of time, ranging from 40 min to a few hours. In certain cases the combustion stays at the smoldering process, in others violent flames appear as a consequence of the smoldering process. Flames can rapidly expand beyond their source and cause serious material damage. Research of different upholstery composites for the furniture industry confirms a significant impact of a defined outer upholstery material on the combustibility of the whole upholstered furniture. This is why attention in the fire protection field should be drawn to increasing the fire resistantce of the outer, textile-covering materials. Agents for the protection of textiles against fire need to have special properties. They must be effective in reducing flammability, resistant to leaching resulting from repeated cleaning, and they should not affect aesthetics, the handle and the drape of the fabric, and meet basic conditions for the protection of the environment and health.

One of the most universal methods for flame-retardancy of textiles, especially for upholstery fabrics, is a back-coating method and the use of it is growing rapidly. In this particular method a flame-retardant composition is applied on the reverse side of the fabric and, therefore, it must be efficient enough to transfer flame-retardant properties to the front side of the fabric, on which an ignition source is applied [1,2,3].

The composition of a coating contains a wide range of chemicals depending on the nature of the polymer. Phosphorus and nitrogen-based systems have turned out to be very effective solutions. Mixtures of these materials may be particularly effective because of additive or synergistic effects [1,3,4,5,6,7]. In recent years, intensified research has been conducted on intumescent flame retardants, which swell as the result of heat or flame and create a thick carbonized and porous insulating layer [1,6,8,9,10]. Studies carried out in this field showed that an intumescent system combined with a fabric gives unexpectedly good fire-retardant and barrier properties. The use of appropriate barriers in upholstered systems reduces the susceptibility of the filling material to the development of fire and its spreading. However, there is a problem of their poor water durability. In order to improve durability, the use of microcapsules of flame retardants as intumescent additives has been studied in textile coatings [8,11]. Additionally, polymer nanoadditives and nanocomposites offer a possibility for the development of a new class of back-coating for textiles [2,12,13,14].

In order to obtain a flame retardant textile back-coating, several flame retardant compositions were prepared and evaluated for: facility of application, appearance of the coating, resistance to leaching, flame-retardant properties, and flexibility of the fabric [15]. As the result of those experiments, a phosphorus-containing flame retardant composition based on siloxane resin and ethylene-vinyl chloride copolymer was obtained (BC).

In this paper the flame retardant properties of the phosphorus-containing composition itself are presented, as well as its effectiveness as a fabric back-coating, and the influence of some additives, (such as fumed silica, montmorillonite, carbon nanotubes and graphite, on its flame-retardant properties) are described. The effect of the novel developed flame-retardant back-coating on the fabric properties, such as flame resistant properties, resistance to washing, antifungal activity, strength and elongation at break, hygroscopicity, and recovery angle are demonstrated.

## 2. Materials and Methods

### 2.1. Materials

Fabrics: linen/cotton fabric—55%/45% with an area density of 360 g/m^2^ (NF) from Runotex S.A. (Kalisz, Poland); velour synthetic fabric—acrylic fleece—65%, polyester/viscose backing—27%/8% with an area density of 444 g/m^2^ (SF) Runotex S.A. Additives: montmorillonite K 10 (MMT) supplied by Aldrich (Steinheim, Germany), multi-walled carbon nanotubes supplied by Nano-Leszek-Partner (Warsaw, Poland), purity over 93%, tube diameter ranges 10 to 40 nm, tube length ranges from 1–25 μm (MWCNT), expandable graphite flake from GrafTech International, Ltd. (Lakewood, OH, USA), Grafguard 160-80A (GR), fumed silica (SiO_2_) with a particle size of 5–50 nm supplied by Wacker Chemie AG (Munich, Germany); phosphorus-containing composition (BC) based on siloxane resin and ethylene-vinyl chloride copolymer, novel flame-retardant fabric back-coating developed as a result of the studies, based on the BC composition with the mixture of different additives, including nanoadditives (EXP).

#### Preparation of Samples

The preparation of flame retardancy compositions was performed using a mechanical stirrer, with the exception of the fumed silica, montmorillonite, and carbon nanotubes, which were introduced into the composition with the use of an ultrasonic processor.

Five parts dry weight of each nano-additive were introduced into 400 parts by wet weight of the BC composition, previously heated to 80 °C, in the case of all of the additives, except for the carbon nanotubes, which was added in the amount of two parts by weight. The obtained compositions, in the form of a paste, were applied on the reverse side of the fabrics in the amount of 350 g wet weight per m^2^. Then the coatings were dried for 5 min at 90 °C and cured for 10 min at 150 °C, using a Mathis Labcoater Dryer with a coating device. Additionally, in order to investigate the effect of additives on the flammability of the BC coating alone (without fabrics), the samples of the obtained paste in the form of films (ca. 1.5 mm thickness) were prepared, dried, and cured at the same temperature and time as the coated fabrics.

### 2.2. Methods

#### 2.2.1. Flammability Methods

- Cone Calorimetry
  Combustibility measurements were performed on a cone calorimeter CONE2A made by Atlas Electric Devices (Chicago, IL, USA). The measurements were carried out in accordance with the ISO 5660-1:2002 standard. The samples were exposed to an external heat flux of 35 kW/m^2^. The orientation of the sample was horizontal, and a spark igniter was used to ignite the combustion gases. Taking into account the application of the fabrics as upholstery, the uncoated outer side of the samples (front face of the fabric) were facing the heater. The heat release rate measurements were taken every 2 s. The fabric samples were cut to the size of 100 mm × 100 mm and wrapped in aluminum foil with the shiny side towards the sample. The BC coating alone and the back-coated fabrics as single layers were tested. During the preliminary tests neither shrinkage nor curling of the samples was observed, therefore, the tests without the use of a wire grid were performed, which is often used in such cases. There are many reports about the cone calorimetric method exploited for textiles, where the influence of a wire grid, grid type, numbers of fabric layers, weight, and thickness of the samples on the flammability results were discussed [16,17,18,19,20,21]. A Superwool^®^ 607 HT blanket (Morgan Thermal Ceramics, Windsor, Berkshire, UK) having a density of 96 kg/m^3^, and a thickness of 13 mm was used underneath aluminum foil to insulate heat transfer from the back of samples. Before the testing, the samples were conditioned for 24 h in a climate chamber at 23 °C and a relative humidity of 50%. The wrapped and conditioned samples were placed in the specimen holder on the four layers of fiber blanket and covered by a retainer frame. The construction of the specimen holder and the retainer frame were in accordance with the ISO 5660 standard. The surface of the samples exposed to the external heat flux was 0.00884 m^2^. The heat release rate (HRR) as a function on time, maximum and average heat release rate (HRR_max_, HRR_av_), time to reach HRR_max_, total heat release (THR) and time to sustained ignition (TTI) were recorded.
- Microcalorimetry Pyrolysis Combustion Flow Calorimetry (PCFC)
  Combustibility of the materials was determined using a Fire Testing Technology Ltd. PCFC instrument (East Grinstead, UK) following the procedure defined in the ASTM D7309-2007a standard. The samples weighing 5 mg (±0.01) were subjected to pyrolysis in the temperature range 75–750 °C at a heating rate 1 °C/s, and then the gaseous pyrolysis products were burnt in a combustor at a temperature 900 °C. The flow was a mixture of O_2_/N_2_ 20/80 cm^3^/min. The heat release temperature (*T*_max_), maximum specific heat release rate (HRR_max_), and time at which HRR_max_ occurs (*t*_max_) were determined.
- Limiting Oxygen Index
  The limiting oxygen index (LOI) was measured according to the Polish Standard PN-76/C-89020. This method allows for determining the minimum concentration of oxygen (in percentage by volume) in the mixture of oxygen and nitrogen, where the sample, mounted vertically in a measuring column, just burns.
- Ignitability of Upholstered Furniture
  The upholstery systems were constructed by combining flame-retardant (FR) fabrics as a covering material and with standard flexible polyurethane foam T-25, with a density of 25 kg/m^3^ as a filling material. Such upholstery systems were subjected to a smouldering cigarette and a match flame equivalent as ignition sources according to EN 1021-1:2014 and EN 1021-2:2014 standards, respectively, to examine the resistance to the ignition and fire barrier effectiveness of back-coated fabrics. The ignition sources were located in the junction between the horizontal seat and vertical backrest surface. If progressive smouldering or flaming of the upholstery is observed within one hour from the beginning of the test, the material does not meet the standard. In the case of the match flame equivalent test, smouldering or flaming are disregarded if they end before 120 s after removal of the burner tube.

#### 2.2.2. Resistance of FR to Washing Out

To determine the resistance of the flame-retardant coatings to washing out, the upholstery systems containing FR-coated fabrics were subjected to multiple washing with warm water (40 °C) and detergents (Karcher RM760 tablets, Winnenden, Germany) by using a spray-suction system (according to Karcher SE 4002 instruction) and evaluated after 1, 5, 10 and 15 wet cleaning cycles. The one RM760 tablet per 5 L of water was used. The cleaning tests were performed by using seat components of chairs in the following combination: upholstery—decorative fabric, flame-retarded on its reverse side, filling—standard polyurethane foam, and a structural component—plywood. The prepared seats were sprayed with detergent solution using a wash nozzle for cleaning upholstery and after 10 min the solvent was sucked. The seats were left to air-dry and then cleaning operations were repeated. After performing a specified number of operations, the upholstery system was disassembled and the fabrics were subjected to the tests.

#### 2.2.3. Antimicrobial Properties of the Fabrics

Determination of the antimicrobial properties of fabrics to the mould action was conducted according to EN 14119:2003 standard. The unprotected and FR-protected fabrics were placed on an agar medium inoculated with a spore suspension of *Aspergillus niger* van Tieghem, allowing the assessment of the fungicidal activity of the back-coated fabrics. The fabric samples (in four repetitions) were placed on an agar medium on the outer side—in this case, the flame-retardant coating was in contact with the agar medium (Z) and the fabric samples (in four repetitions) were also placed on an agar medium on the agar side—in that case the flame-retardant coating was not in direct contact with the agar medium (W). Incubation of fabric samples in a temperature 29 ± 1 °C and relative air humidity at 90% was conducted for 14 days. After the tests, evaluation of antimicrobial properties was performed on the basis of visual assessment by determination of the fungus growth degree on the surface of the fabrics (outer side and agar side) according to a five-grade scale, as follows: 0—no visible growth evaluated microscopically; 1—no visible growth evaluated with the naked eye, but clearly visible microscopically; 2—growth visible with the naked eye, covering up to 25% of the tested surface; 3—growth visible with the naked eye, covering up to 50% of the tested surface; 4—considerable growth, covering more than 50% of the tested surface; 5—very intense growth, covering all of the tested surface.

#### 2.2.4. Utility Properties

Determination of tensile strength and elongation at break were performed according to PN-EN ISO 1421:2001 standard using Automatic Tensile Tester STATIMAT ME (Textechno H. Stein GmbH and Co. KG, Mönchengladbach, Germany). The property of the coated fabrics to recover from creases by the measurement of the recovery angle were determined in accordance with the PN-EN 22313:2000 standard on a Crease Recovery Tester of Woven Fabrics, Monsant Model MR-1 (Daiei Kagaku Seiki Mfg. Co., Ltd., Kyoto, Japan). Additionally, hygroscopicity of fabrics at 65% and 100% relative humidity of air were investigated according to PN-P-04635:1980 standard (weight method).

## 3. Results and Discussion

### 3.1. Flammability Results of the BC Composition without and with Different Additives

#### 3.1.1. Results from Cone Calorimetry

**Figure 1:**
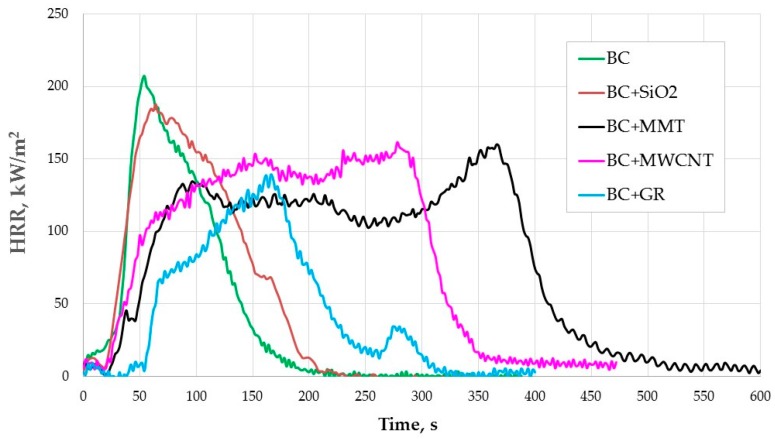
Cone calorimeter HRR for the modified BC composition coating alone with different additives.

Figure 1 and Table 1 present flammability parameters of the BC composition with and without additives tested on the cone calorimeter.

Graphite shows extraordinary efficiency in additional reduction of flammability of the BC composition. It reduces HRR_max_ in the highest degree (i.e., by 35%) and extends the time to ignition of the BC composition two-fold (Table 1). Total heat release rate (THR) is comparable to the base composition. Average heat release rate (HRR_av_) increased as a result of adding graphite to the composition only to a small degree. Graphite had also a significant impact on extending the time to HRR_max_ (from 54 to 167 s). This is due to the fact that at high temperatures graphite oxidizes and releases gases, which causes the expansion of the material. A porous layer made of expanded graphite lamella suffocates the flame and acts as a physical barrier for heat and mass transfer [9,22].

The carbon nanotubes (MWCNT) and montmorillonite (MMT) additions were the most effective in delaying time to reach HRR_max_. The combustion process of the BC composition changed after introduction of MWCNT and MMT, what can be seen in the Figure 1 and Table 1.

The HRR curve for BC composition with MMT (BC + MMT) is characterized by two clear peaks of HRR, between which there is a plateau period of continuous combustion at about 120 kW/m^2^. The sample burns twice as long. MMT reduces significantly HRR_max_ i.e., by 24.3% and prolongs the time to HRR_max_ by more than seven times, as HRR_max_ occurs after a long plateau at the second HRR peak. MMT slightly prolongs the time to ignition of the BC composition (by about 4 s).

Additionally, in the case of using MWCNT in the BC composition the HRR curve (Figure 1) was characterized with a plateau, but at a higher HRR level as compared with BC + MMT (at 130–150 kW/m^2^). Then a slight increase of HRR was observed until it reached HRR_max_, 21.0% lower than HRR for the BC composition. The time to ignition in the case of the BC and BC + MWCNT compositions was similar.

However, MMT and MWCNT have a significant effect on the increase of HRR_av_ and THR of the BC composition, as those additives significantly prolong the combustion time of this composition, with a plateau period observed at a relatively high temperature. In the case of BC + MWCNT the burning time is about 350 s, for BC + MMT is about 500 s and for the BC coating alone it is only about 200 s. This difference is due to the fact that the addition of both the MWCNT and MMT contribute to the formation of protective layer barrier structures against heat and volatiles during the combustion [23,24,25,26,27]. Their degradation takes much more energy (compared to the BC alone and with other additive compositions) and, thus, the time due to breaking of a large number of bonds that are between the atoms forming the structures [28]. Experimental results reported in the literature showed slow burning for a longer time of the nanocomposites, which significantly reduced the peak heat release and a decreased mass loss rate, but without improvement of the total heat release rate [29,30,31,32,33,34,35,36]. A more detailed characterization of the BC composition with these nanoadditives is needed in further studies to understand their impact on the heat release rate by doing thermogravimetric analysis, Fourier transform infrared spectroscopy analysis of combustion residues, morphology, and also the mechanical properties. Many previous studies have shown thermal stability and flammability characteristics of montmorillonite and carbon nanotube-polymer nanocomposites and several mechanisms have been proposed to describe the flame-retardant properties of these nanocomposites [2,25,26,27,28,29,30,31,32,33,34,35,36,37,38,39,40,41,42,43,44]. However, only limited studies on the impact of the montmorillonite and carbon nanotubes on the total heat release rate of polymers have been reported.

Fumed silica introduced to the BC composition shows the least efficient activity among the tested additives, in terms of reducing flammability. SiO_2_ lowers the HRR peak of the BC composition only slightly in comparison with MMT and MWCNT. Fumed silica also has the weakest effect on prolongation of time to HRR_max_ and shortens the time to ignition of the BC composition.

##### Back-Coated Fabric Flammability

The front face of the fabric was exposed to the heat flux in cone calorimeter. That side was not FR coated.

**Figure 2:**
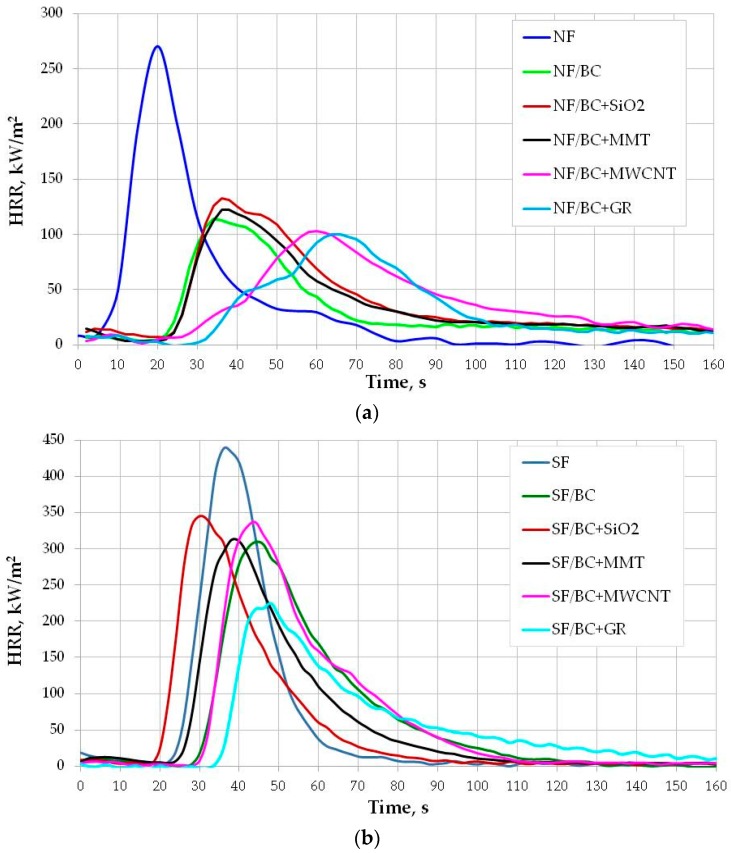
Cone calorimeter HRR for the fabrics back-coated by the BC composition with different additives: (**a**) natural fabrics; and (**b**) synthetic fabrics.

Flammability parameters of the fabrics back-coated by the BC composition with and without different additives determined from the cone calorimeter are presented in the Figure 2 and Table 1.

The curves of heat release rate for the fabrics with coatings containing MMT and MWCNT (Figure 2) are different than HRR curves characteristic for the BC coating alone with these additives (Figure 1). For the treated fabrics, a single distinct HRR peak was observed without the plateau. In the case of the BC coating with MMT and MWCNT, a long period plateau was observed between two HRR peaks. The combustion process of the fabrics, themselves, is a key factor determining the combustion of the fabrics with the back-coating in the cone calorimeter test.

All of the applied coatings efficiently decrease the flammability of the natural fabric (NF). Figure 2a clearly shows the significantly reduced HRR peak (characteristic for the natural fabric) after treatment with FR coatings, the longer time to reach HRR_max_, and also the time to ignition of the fabric. The effect of the applied compounds on the flammability parameters of the synthetic fabric (SF) depends on the type of the additive; only in the case of HRR_max_ do all of the compositions show the same effect i.e., they reduce HRR_max_.

The BC composition used as back-coating for the natural fabric has the effect of reducing HRR_max_ by 56.8%, and the time to reach HRR_max_ increases from about 21 to 35 s (Table 1). Ignition of the back-coated fabric followed 10 s later compared to the uncoated fabric. The BC coating also slightly reduced HRR_av_ and THR. Additionally, in the case of the synthetic fabric, the BC coating, apart from lowering HRR_max_ (by about 28.3%), prolonged the time to HRR_max_ and TTI, but to a lesser extent compared to the natural fabric. THR and HRR_av_ increased marginally after treatment of the SF fabric by the BC back-coating.

Considering that the fabrics were coated only on the reverse side and the unprotected front surface of the fabric was exposed to the heat flux, the fire-retardant action of the composition is especially effective. The formation of char occurs because of the reverse side of the fabric and the char must be sufficiently effective to prevent burning of the front of the fabrics [45]. The BC coating is particularly effective in the case of natural fabrics due to phosphates present in the composition, which forms polyphosphoric acid that leads to dehydration of the cellulose in the fabric and the formation of char.

Introduction of the additives to the BC composition has different effects on the fabric flammability and depends not only on the type of the additive but also on the type of the fabric.

Based on a comparison of the flammability parameters of the fabrics coated with the BC composition without and with GR, MMT, SiO_2_, and MWCNT (Figure 2), it can be stated that graphite is the most effective in promoting further reduction of flammability both for natural and synthetic fabrics. It has the strongest influence on improvement of such parameters as HRR_max_, time to HRR_max_, and also TTI, with the exception of the TTI of NF/BC + MWCNT, which is characterized with longer TTI than NF/BC + GR. MWCNT turned out to be very efficient in delaying ignition of the natural fabric. Carbon nanotubes also extended the time to ignition of synthetic fabric but to a lesser extent. The addition of MWCNT to the BC coating has an effect on further lowering of HRR_max_ and time to reach HRR_max_ of the natural fabric.

From the tested additives, fumed silica turned out to be the least efficient in improving flammability parameters of the fabrics coated with the BC composition. The fabrics, both natural and synthetic, coated with BC + SiO_2_ composition showed the highest HRR_max_ as compared with the fabric coated with the BC coating without SiO_2_. The data in Table 1 indicates the much weaker FR activity of the BC + SiO_2_ coating on the synthetic fabric. The addition of fumed silica in this case results in shortening the time to HRR_max_ and TTI, not only for the fabric with BC composition but also for the untreated fabric. The weaker effect of SiO_2_ in reducing fabric flammability is reflected in its similarly poor effect for the BC coating alone. Introduction of the fumed silica to the very effective flame-retardant coating lowered its efficiency. Horrocks et al. also showed that the addition of fumed silica to a flame-retardant back-coating reduces its effectiveness [46]. However, previously our research showed that the application of nanosilica in intumescent systems increases the efficiency of fireproofing properties of systems with respect to those of wood. A high degree of particle dispersion causes changes in combustion processes, as well as decomposition of intumescent coating by modifying the carbon structure into a small-cell one, which improves thermo-insulating properties, conductivity, and heat convection of the foam formed [47]. However, Wang et al. concluded that the well-distributed nano-SiO_2_ particles in an acrylic nanocomposite could modify char formation and anti-oxidation of char structure at high temperature [48].

Regarding the effect of montmorillonite as an additive to the BC composition, it can be seen in Figure 2 and Table 1 that the fabrics, both the natural and synthetic ones, back-coated with BC + MMT composition (NF/BC + MMT, SF/BC + MMT) are characterized with higher HRR_max_ in comparison with the fabrics treated with the BC back-coating alone (NF/BC), SF/BC). The literature also reports that the nanoparticle clays have no beneficial effect as additives to back-coating compositions [45]. Considering other flammability parameters, it seems that MMT action is more efficient for the synthetic fabric (Table 1). The addition of MMT had an effect on further lowering of HRR_av_ and THR in the synthetic fabric (comparison of SF/BC and SF/BC + MMT). It must be mentioned that MMT increased the values of those parameters for the BC alone (BC and BC + MMT). This results from, as mentioned earlier, the influence of the fabric ignition geometry (testing fabrics on the uncoated side) on the processes occurring during combustion of the fabrics treated with BC coating with MMT and, consequently, from the presence of a single peak in the HRR curve.

#### 3.1.2. Results from PCFC Microcalorimetry

**Figure 3:**
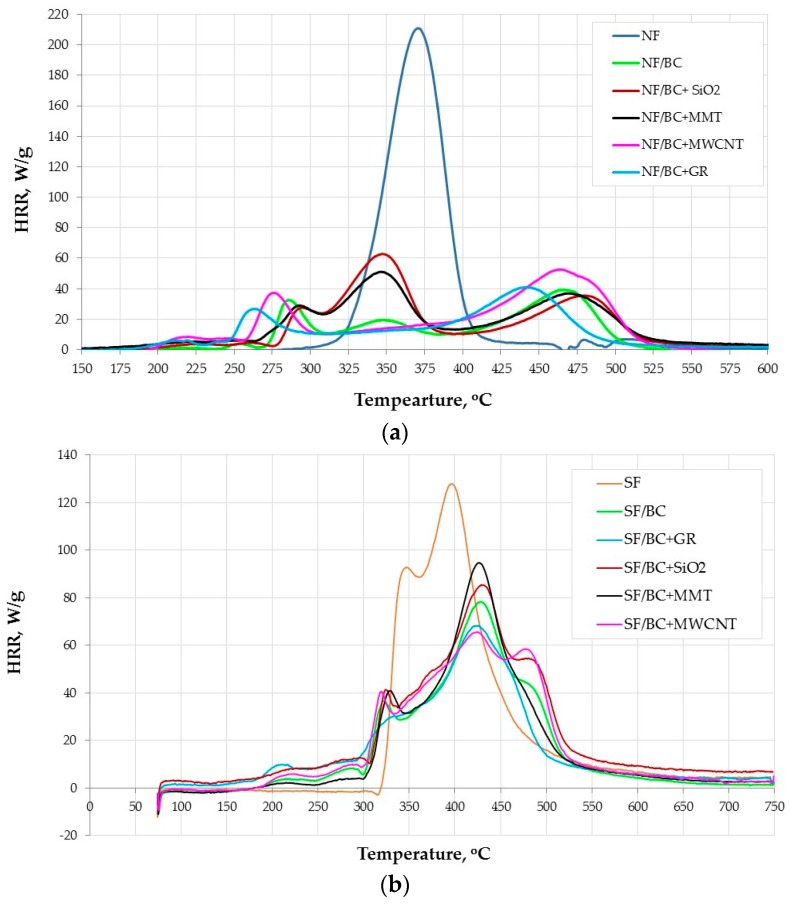
PCFC HRR for the fabrics back-coated by the BC composition with different additives: (**a**) natural fabrics; and (**b**) synthetic fabrics.

The heat release rate curves for the uncoated natural and synthetic fabrics and those back-coated with different compositions are shown in Figure 3, and the heat release temperature (*T*_max_), maximum specific heat release rate (HRR_max_), and time at which HRR_max_ occurs (*t*_max_) are presented in Table 2.

It is evident that the HRR curves for the fabric differ between the cone calorimeter and PCFC techniques (Figure 2).

It is clearly evident that the HRR curves for the fabrics differ between the cone calorimeter and PCFC techniques (Figure 2). During combustion in the cone calorimeter the HRR grows, reaching a single distinct maximum, while for the PCFC we observe more stages of the decomposition and combustion, what is reflected in two or more HRR peaks on the curves, depending on the type of the introduced additive to the base composition. These different behaviours of fabrics are affected by many factors. Firstly, the cone calorimeter represents a well-defined flaming condition, forced by external heat flux and a spark igniter for igniting the combustion gases, typical for a developing fire scenario [48], whereas PCFC is a non-flaming test, which separately simulates solid-state phase pyrolysis by heating in an inert gas stream and subsequent gas phase processes (combustion) by rapid oxidation at high temperature, in excess oxygen of volatile pyrolysis products [49]. In addition, as previously mentioned, in the cone calorimeter test the outer surface of the fabrics (100 mm × 100 mm) was exposed to the heat flux, whereas in the PCFC the samples of different size, shape, orientation and weight (5 mg) were tested. This is particularly important in the case of testing fabrics with flame retardants and in addition in the form of back-coatings (i.e., heterogeneous samples).

Similar HRR curves from cone calorimetry and PCFC (a single HRR peak) are observed only for the natural fabric (Figure 3a), while at the curve for the synthetic fabric (Figure 3b) two peaks of HRR are observed, due to the layered construction of the fabric (fleece and backing) and various kinds of synthetic fibers.

The BC coating reduces HRR_max_ of the natural fabric by more than 80%, prolongs the time to reach HRR_max_ by 93 s and raises HRR_max_ temperature by 98 °C. The maximum HRR falls for the third HRR peak. The first peak of HRR of about 33 W/g occurs at 285 °C after 230 s, then HRR is reduced to about 15 W/g. This results from effective action of the BC composition. The use of phosphorus and nitrogen flame retardants in the composition, whose elements strongly interact with cellulose, leading to intensive forming of char, releasing non-flammable gases, which dilute the flammable gaseous products of combustion. Additionally, the intumescent agent present in the coating, which forms a phosphoro-carbonaceous structure, efficiently protects the material [25]. These different reactions occur during this stage at a different time. At about 350 °C a slight decrease of FR action happens resulting in an increase of the heat release rate (as the very small second HRR peak). When the fire protective action of char fades, the HRR grows and after 412 s it reaches the third peak, slightly higher than the first one (39.4 W/g).

Regarding the effect of the additives introduced to the BC composition on improving its fire retardant efficiency, fumed silica and montmorillonite turned out to be of low efficiency (Figure 3, Table 2), which confirms the results obtained by the cone calorimetric method. The decomposition and combustion processes of NF/BC + MMT and NF/BC + SO_2_ proceed in a very similar way. They reach HRR_max_ at the same time and similar temperature, where the fabric back-coated with MMT shows a slightly lower HRR_max_ in comparison with SiO_2_. MMT and SiO_2_ reduce the intensity of heat release at the beginning of decomposition and combustion of the natural fabric—namely before the first HRR peak. Their influence however, changes the combustion process of the BC back-coated NF fabric so that the HRR_max_ values fall for the second HRR peak, and they reduce the time and temperature at which the HRR reaches the maximum, and HRR_max_ values are about 1.5 times higher compared to NF/BC.

In general, the natural fabrics back-coated with the BC composition separately containing the tested nano-additives are characterized with higher values of HRR_max_ compared to the fabric with only the BC coating. In cone calorimetry the MWCNT and GR lowered that parameter, but not significantly.

The HRR curves for NF/BC + MWCNT and NF/BC + GR are characterized with only two peaks. The first HRR peak in case of the MWCNT and GR occurs at lower temperature (about 275 and 265 °C, respectively) than in the case of the BC coating, whereas the peak value is lower after introduction of the graphite and higher when the nanotubes are present. The efficient action of the graphite and nanotubes is clearly visible in Figure 3a, where they caused the elimination of the second HRR peak present in the curve characteristic for NF/BC. In the curves for NF/BC + GR and NF/BC + MWCNT, between the first and second peak, there is a clear reduction of HRR below the curve for the NF/BC and very slow growth of HRR until reaching the second peak, with slightly higher values than in the case of the NF/BC sample (about 5–15 W/g). It is interesting that the trends of heat release for the natural fabric treated with these two compositions are very similar to those observed for the HRR curves for the BC + MWCNT and BC + GR from the cone calorimetry (Figure 1).

When present on the synthetic fabric the PCFC data shows, that the BC coating reduces HRR_max_ by about 40%, delays the time to HRR_max_ by 30 s and increases the HRR_max_ temperature by 30 °C. The HRR curves of the coated fabric have three peaks, where the HRR_max_ values are reduced for the second HRR peak.

Yet, a distinct difference can be observed in efficiency of the BC coating between the natural and synthetic fabrics. In the case of the natural fabric, the protective action of formed char is more intense and effective. As showed in Figure 3a, at temperatures >400 °C when the natural fabric had undergone complete degradation/combustion (curve NF); for the BC back-coated natural fabric the fire-retardant action still continued, while the range of the decomposition and combustion temperature of the SF and the BC back-coated SF fabrics are similar.

Additionally, for the synthetic fabric, MMT and SiO_2_ do not affect further reductions in HRR_max_ after adding them to the BC composition. On the contrary, HRR_max_ increased by 20% and 15%, respectively. It is worth mentioning that the montmorillonite shows the better properties in reducing the intensity of heat release after the first peak (between about 320 and 390 °C) compared to the SiO_2_, and also MWCNT and the lowering of the HRR is comparable with the HRR reduction in the case of the BC coating.

It is interesting that coating with the MWCNT has the strongest effect among the tested compositions on lowering the HRR_max_ of the synthetic fabric (by 50%) and, with respect to the BC coating on the SF fabric, it additionally reduces HRR_max_ by about 16%. The data from PCFC suggests a considerable effect of the MWCNT in the strengthening of char formation, probably also by the interaction of nanotubes with phosphorus compounds, thus delaying intensive combustion [12].

The efficient action, discussed earlier, of the graphite in improvement of flame retardancy properties of the BC composition was also confirmed under the PCFC conditions for the synthetic fabric. The HRR curve for SF/BC + GR does not have a first peak, and so is distinct from other compositions and HRR_max_ for the SF fabric with the BC coating, being reduced by another 10% after the introduction of the graphite.

#### 3.1.3. Limiting Oxygen Index

**Figure 4:**
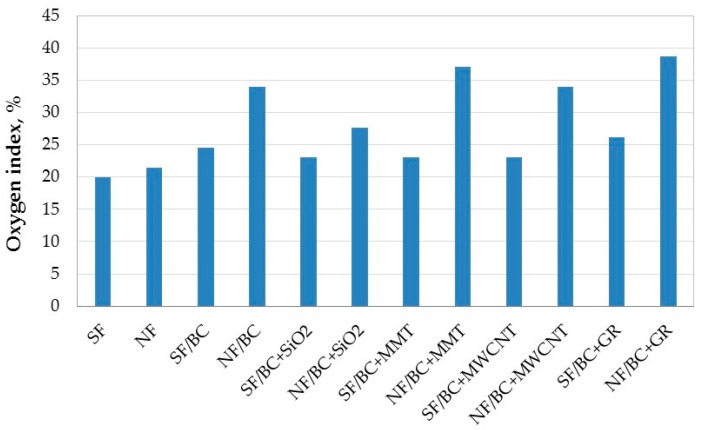
Oxygen index for the natural (NF) and synthetic (SF) fabrics back-coated by the BC composition with different additives.

In Figure 4 we can observe improvement of the oxygen index for the back-coated fabrics compared to the unprotected fabrics.

The BC back-coating increased the LOI value of the natural fabric from 21.4% to 34.0%, and of the synthetic fabric from 19.9% to 24.6%.

The effect of additives is greater in influencing the flame-retardant action of the natural fabric compared to the synthetic fabric, and generally the LOI values are much higher.

The most significant increase of LOI of the natural and synthetic fabrics is observed for the fabrics coated with the BC composition containing graphite (38.7% and 26.1% respectively).

LOI values of the synthetic fabrics show that fumed silica, carbon nanotubes, and montmorillonite show similar effects on the LOI values and do not improve the fire properties of the BC coated SF fabric. When they are added to the BC composition the LOI value decreases from 24.6% to 23.0%.

The natural fabrics back-coated with the BC composition containing the montmorillonite show their resistance to burning with higher LOI values (37.1%) compared to the NF fabric coated with the BC coating. The addition of carbon nanotubes does not further change the LOI value (34.0%), and fumed silica decreases flame-retardant action of the BC coating (27.7%).

### 3.2. The Effect of the Novel Flame Retardant Back-Coating (EXP) on the Properties of the Synthetic Fabric before and after Multiple Wet Cleaning

#### 3.2.1. The Flame Retardant Effectiveness

**Figure 5:**
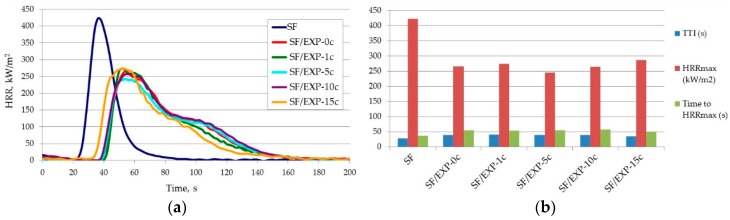
Cone calorimeter results for the unprotected and the EXP flame-retardant back-coated synthetic fabric before and after multiple wet cleaning: (**a**) HRR as a function of time; and (**b**) TTI, HRR_max_ and time to reach HRR_max_.

High flame-retardancy effectiveness of the novel flame-retardant coatings (EXP) was confirmed by cone calorimetry results both before and after multiple washing (Figure 5). FR coatings were found to protect the synthetic fabric by delaying ignition and time to reach HRR_max_, and by reducing the rate of released heat and maximum heat release rate (HRR_max_).

**Figure 6:**
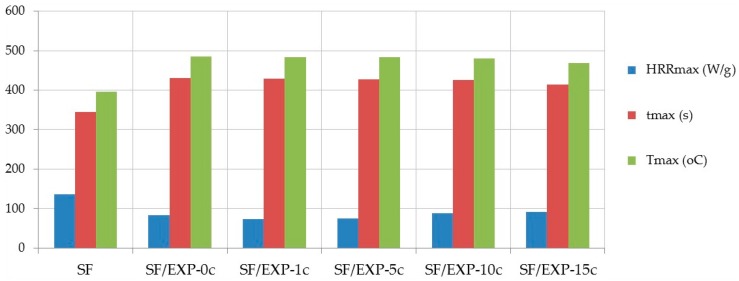
PCFC results for the EXP flame-retardant back-coated synthetic fabric before and after multiple wet cleaning.

The PCFC results confirm the reduction in combustion intensity of the fabric back-coated by the EXP flame retardant. The coating significantly decreases HRR_max_, and increases temperature (*T*_max_) and time (*t*_max_) to reach the HRR_max_ (Figure 6). After 15 washing cycles all of the parameters are only slightly worse than those of the coated fabric before washing (about 4%).

**Figure 7:**
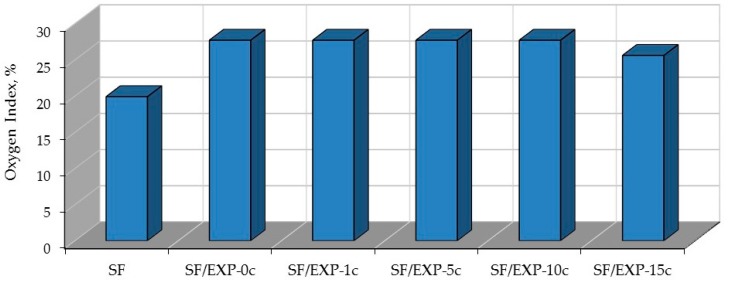
Oxygen index for the the EXP flame-retardant back-coated synthetic fabric before and after multiple wet cleaning.

The novel flame-retardant coating increases the oxygen index by about 40% (Figure 7). Even 10 washing cycles do not change the oxygen index value. Only after 15 washing cycles slight decreasing of the LOI value is observed.

The ignitability results before and after multiple cleaning of upholstered furniture with the unprotected fabric and protected by the EXP flame-retardant back-coating tested according to EN 1021 are presented in Table 3.

The result showed in Table 3 indicate that the unprotected and the EXP back-coated fabrics pass the smoldering ignition test (ignition by cigarettes), the fabrics do not ignite and smolder. The EXP coated fabrics, before and after multiple cleaning by the spray-suction system (up to 15 cycles), also pass the flame ignition test (simulated match) in contrast to an unprotected fabric, which ignite during the test.

#### 3.2.2. Utility Properties of the Fabric Back-Coated with the EXP Flame Retardant

The fabric back-coated with the EXP flame retardant have a higher breaking strength and elongation at break both before and after 15 washing cycles; also hygroscopicity and relaxation angle are slightly different compared to the unprotected fabric (Table 4).

#### 3.2.3. Antimicrobial Properties of the Synthetic Fabric Back-Coated with the EXP Flame Retardant

Table 5 shows the results of tests for antimicrobial properties of the synthetic fabric back-coated with the EXP flame retardant.

**Figure 8:**
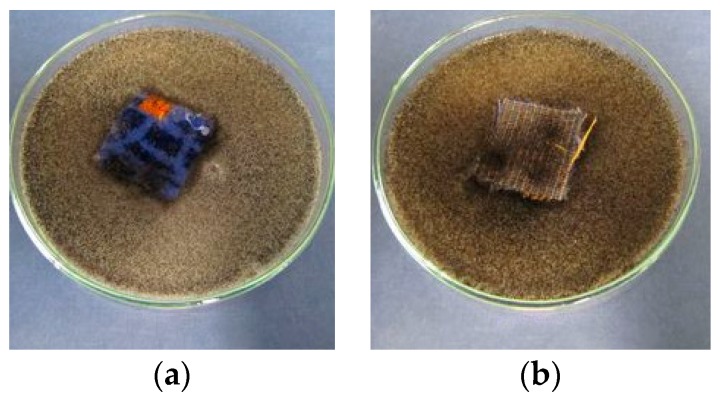
The control sample of the uncoated SF fabric, exposed to the action of a fungus *Aspergillus niger*: (**a**) outer side; and (**b**) agar side.

The control samples of the uncoated fabric showed lowered resistance to the action of *Aspergillus Niger* fungi. On the outer side of the tested samples 2° was observed, i.e., the fungi growth was visible with a naked eye, covering up to 25% of the tested surface, while on the agar side 4° was observed, i.e., severe fungi growth, covering more than 50% of the tested surface (Figure 8).

**Figure 9:**
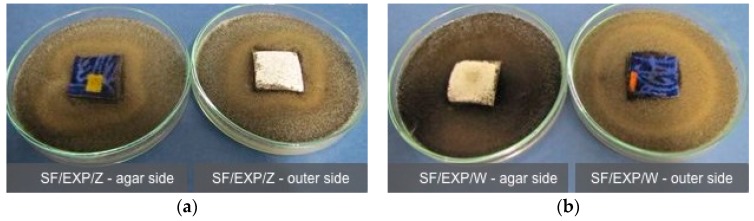
The synthetic fabric back-coated with the EXP flame retardant, exposed to the action of a fungus *Aspergillus niger*: (**a**) SF/EXP/Z; and (**b**) SF/EXP/W.

In the case of the fabric protected with the EXP coating limited fungi growth was observed, both in the samples and in the agar medium, in the zone of direct contact with the samples. On the sample surface, at the sides with the EXP coating and the untreated one, regardless of their exposure (outer or agar side), 2° growth was observed, i.e., the tested fungi was visible with a naked eye and it did not cover more than 25% of the sample surface (Figure 9). The fabric back-coated with the new FR exhibited a higher degree of antifungal activity as compared to the unprotected fabric.

## 4. Conclusions

All of the tested coating compositions based on siloxane resin and ethylene-vinyl chloride copolymer are very effective in conferring flame retardancy on the fabrics. Furthermore, all of the tested additives turned out to be very effective in reducing the maximum HRR, the time to reach maximum HRR, and the ignition time (with the exception of the fumed silica) of the BC coating alone. However, MMT and MWCNT have a significant effect on the increase of the average HRR and the total heat release of the BC coating alone.

This study also shows the crucial role that the kind of fabrics can play in the flame retardancy effectiveness of the back-coating composition. Generally, the flame-retardant action of the coatings is stronger with respect to the natural fabric in comparison with the synthetic fabric.

The impact of particular additives introduced to the composition based on siloxane resin and ethylene-vinyl chloride copolymer to improve its flame retardant properties depends on the flammability test conditions. Not only have the parameters regarding the cone calorimeter and microcalorimeter affected the combustion studies, but also the different constructions of the natural and synthetic fabrics and flame-retardant method—applying of the coating on the reverse side of the fabrics. These factors explain why the flammability results from these two test methods do not agree in many cases.

Generally, it can be concluded that the addition of graphite has an extraordinary impact in additionally reducing flammability of the coating composition alone. It is also the most effective in reduction of flammability both for the natural and synthetic fabric as an additive in the back-coating compositions. The fumed silica introduced to the tested coating composition shows the least efficient activity, among the tested additives, in terms of reducing the flammability of the coating alone, and back-coated fabrics. The montmorillonite clay is more effective in improving flammability properties of the coating alone compared to the carbon nanotubes, however, the trend is reversed when applied in the back-coating of fabrics. The multiwalled carbon nanotubes turned out to be very effective as additives for the coating in reducing flammability of the natural fabric only.

The novel flame-retardant coating based on siloxane resin and ethylene-vinyl chloride copolymer with different flame-retardant additives is also resistant to multiple cycles of cleaning. The flame-retardant coating improves the mechanical properties of the fabric, while maintaining its aesthetic qualities. The fabrics back-coated with the new flame retardant exhibits a higher degree of antifungal activity as compared to the unprotected fabrics.

## Figures and Tables

**Table 1:** Cone calorimeter combustion parameters for the modified BC composition coating alone with additives and back-coated natural (NF) and synthetic (SF) fabrics.

Composition	HRR_max_ kW/m^2^	Time to HRR_max_, s	TTI, s	HRR_av_ kW/m^2^	THR, MJ/m^2^
BC	209.6	54	25	61.5	16.5
BC + SiO_2_	190.4	81	22	71.0	20.3
BC + MMT	158.6	367	28	95.2	46.0
BC + MWCNT	165.6	279	25	108.1	35.3
BC + GR	139.2	167	57	67.3	17.3
NF	270.8	21	13	20.1	6.1
NF/BC	117.0	35	24	19.3	5.8
NF/BC + SiO_2_	135.1	36	26	25.6	7.3
NF/BC + MMT	125.8	38	25	23.4	6.7
NF/BC + MWCNT	111.6	61	42	25.9	7.5
NF/BC + GR	106.7	64	36	19.7	5.9
SF	438.8	37	27	31.2	9.4
SF/BC	314.6	45	30	35.2	10.4
SF/BC + SiO_2_	345.3	31	26	32.9	9.4
SF/BC + MMT	321.2	40	27	33.4	9.7
SF/BC + MWCNT	339.7	44	32	39.2	10.8
SF/BC + GR	224.6	49	40	33.2	9.0

**Table 2:** PCFC flammability parameters for the natural (NF) and synthetic (SF) fabrics back-coated by the BC composition with different additives.

Composition	*t*_max_ (s)	*T*_max_ (°C)	HRR_max_ (W/g)
NF	320	370	210.9
NF/BC	412	468	39.4
NF/BC + SiO_2_	293	346	63.9
NF/BC + MMT	293	347	51.2
NF/BC + MWCNT	407	464	53.0
NF/BC + GR	390	445	42.1
SF	345	397	128.0
SF/BC	371	428	78.7
SF/BC + SiO_2_	375	430	90.0
SF/BC + MMT	374	427	94.8
SF/BC + MWCNT	368	423	65.9
SF/BC + GR	366	421	70.6

**Table 3:** Ignitability of upholstered furniture with the EXP flame-retardant back-coated synthetic fabric before and after multiple wet cleaning.

Sample	EN 1021-1	EN 1021-2
SF	Passes the test	Does not pass the test
SF/EXP-0c	Passes the test	Passes the test
SF/EXP-1c	Passes the test	Passes the test
SF/EXP-5c	Passes the test	Passes the test
SF/EXP-10c	Passes the test	Passes the test
SF/EXP-15c	Passes the test	Passes the test

**Table 4:** Utility properties of the EXP flame-retardant back-coated synthetic fabric before and after multiple wet cleaning.

Sample	Breaking force (N)	Standard deviation.	Elongation at break (%)	Standard deviation	Hygroscopicity (%)		Angle relaxation (°)	
					65%	100%	Outer side	Reverse side
SF	889	5.90	22.5	2.93	1.14	1.22	157	93
SF/EXP-0c	999	8.04	24.6	1.97	2.97	6.17	138	104
SF/EXP-10c	1,072	1.15	25.7	1.57	2.77	5.87	137	107
SF/EXP-15c	1,043	3.33	26.0	5.13	1.89	3.98	137	105

**Table 5:** Evaluation of the growth of the fungus *Aspergillus niger* on the unprotected and the EXP flame-retardant back-coated synthetic fabric.

Sample	Degree of fungus growth on the fabric	Degree of fungus growth in agar medium in the contact zone with the fabric
SF—Control (outer side/agar side)	2°/4°	5°/4°
SF/EXP/Z—(outer side/agar side)	2°/2°	5°/2°
SF/EXP/W—(agar side/outer side)	2°/2°	5°/2°
